# Supplementary material for: Does TBC1D4 (AS160) or TBC1D1 Deficiency Affect the Expression of Fatty Acid Handling Proteins in the Adipocytes Differentiated from Human Adipose-Derived Mesenchymal Stem Cells (ADMSCs) Obtained from Subcutaneous and Visceral Fat Depots?
Source: Cells. 2021 Jun 16;10(6):1515. doi: 10.3390/cells10061515 (PMC8235367; doi:10.3390/cells10061515)
Supplement: Supplementary file 1 [file cells-10-01515-s001.zip › cells-1237567-supplementary.pdf]

Supplementary Materials:

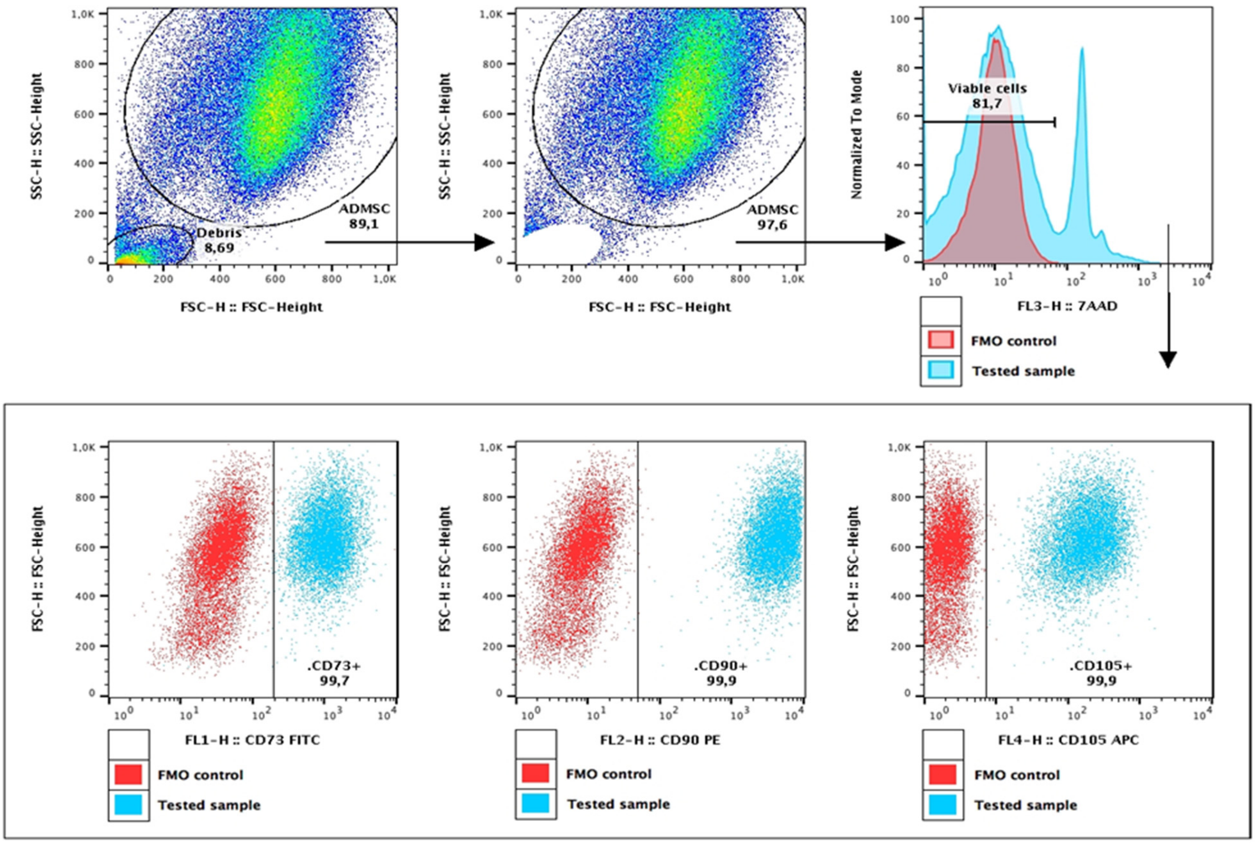

Figure S1. Gating strategy for ADMSCs stem cells derived from visceral and subcutaneous adipose tissues.

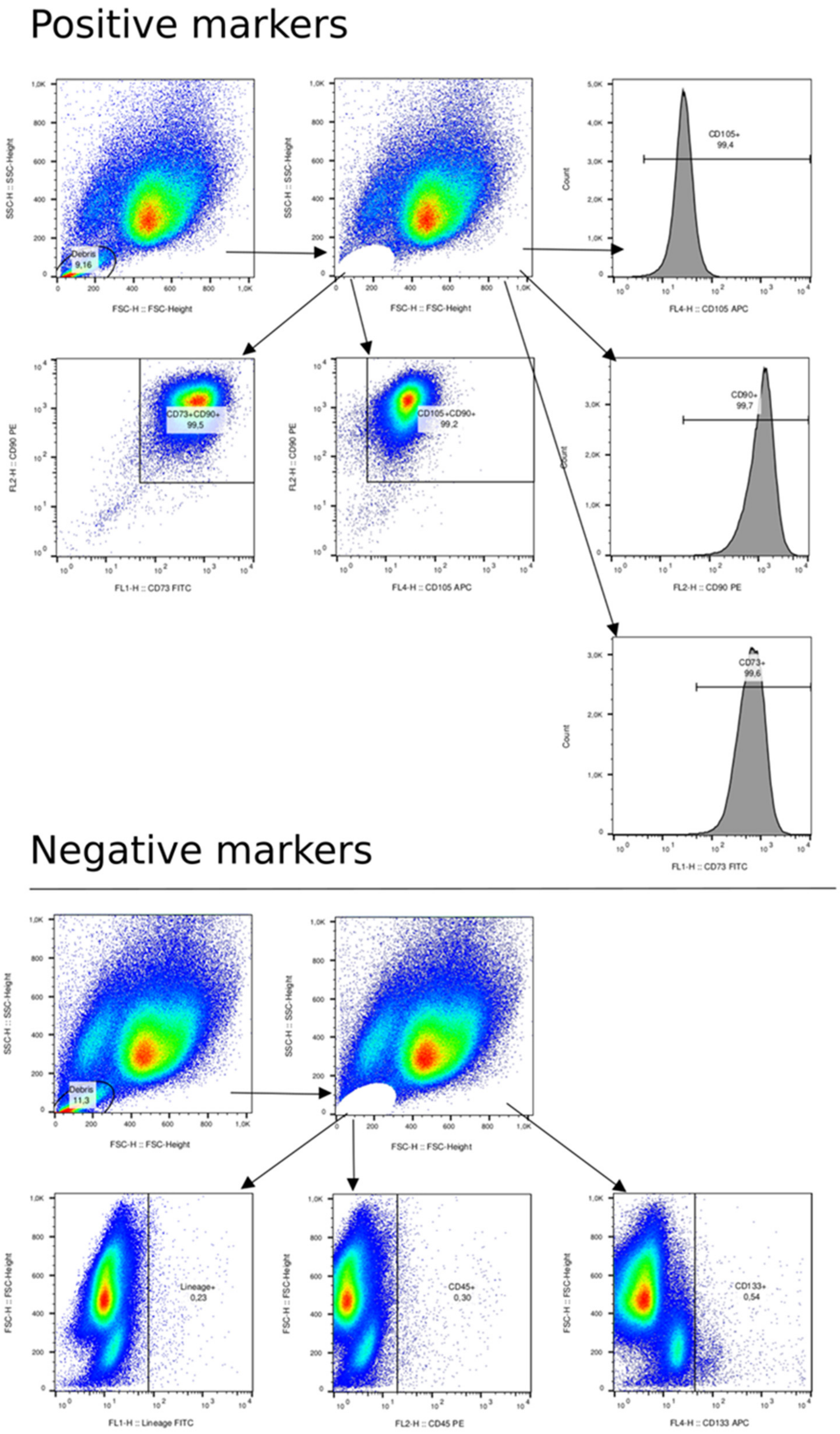

Figure S2. Surface markers of ADMSCs determined by flow cytometry. Numbers indicate the percentage of stained cells in the population compared with the unstained control. Percentage of positive cells are shown each peak for markers CD105, CD73, CD90 and for the cell lacking the negative containing hematopoietic markers (e.g., CD45).

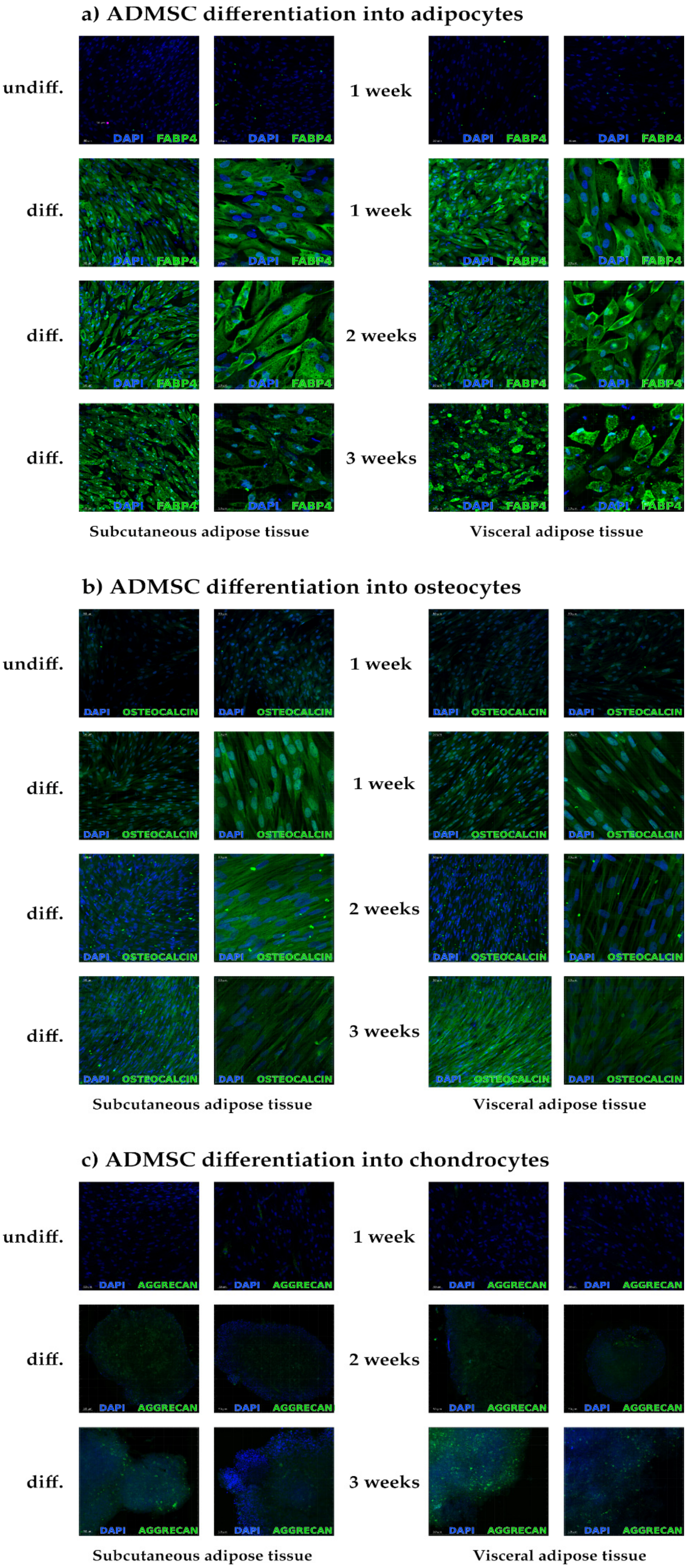
a) ADMSC differentiation into adipocytes
undiff.
1 week
diff.
1 week
diff.
2 weeks
diff.
3 weeks
DAPI FABP4
Subcutaneous adipose tissue
Visceral adipose tissue
b) ADMSC differentiation into osteocytes
undiff.
1 week
diff.
1 week
diff.
2 weeks
diff.
3 weeks
DAPI OSTEOCALCIN
Subcutaneous adipose tissue
Visceral adipose tissue
c) ADMSC differentiation into chondrocytes
undiff.
1 week
diff.
2 weeks
diff.
3 weeks
DAPI AGGRECAN
Subcutaneous adipose tissue
Visceral adipose tissue

Figure S3. Adipogenic (**a**), osteogenic (**b**) and chondrogenic (**c**) differentiation of ADMSCs. Immunostaining of FABP4, osteocalcin and aggrecan (green) and nuclei (blue) was performed. The process of multilineage differentiation of ADMSCs was weekly monitored for the three weeks. Magnification 20x for the left and medium images and 60 x for the right side of the figure. Scale bars 50 μm.

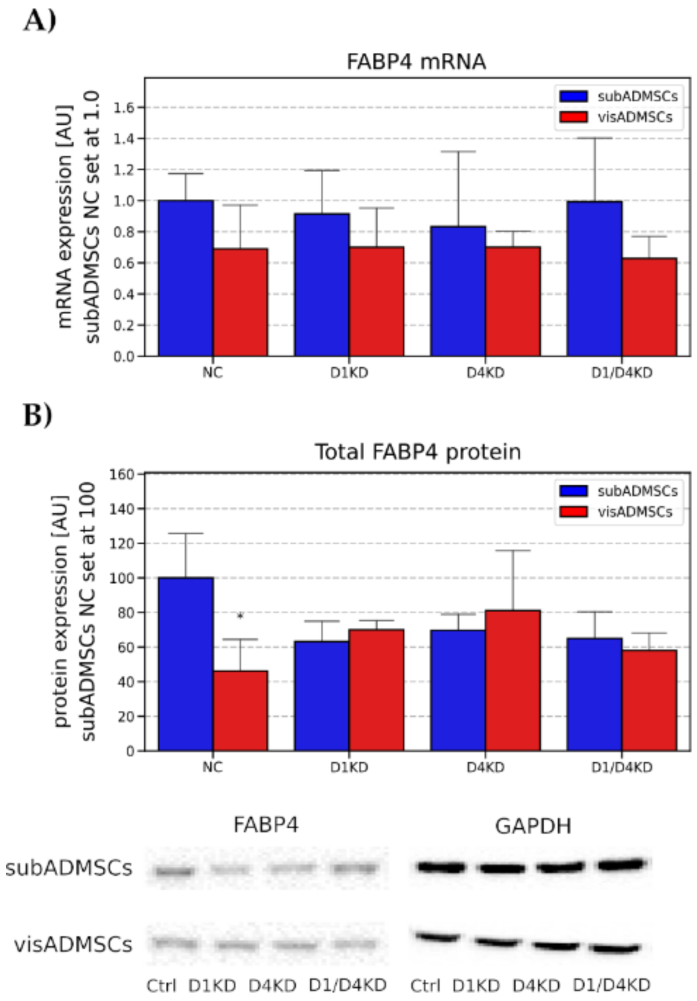

Figure S4. FABP4 mRNA (**A**) and protein (**B**) abundance in TBC1D1 and/or TBC1D4 deficient adipocytes. Error bars represent mean +/−SD. Number of patients equals 4 (measurements taken in duplicate). Values are expressed in arbitrary units; subADMSCs control group was set as 100. Representative Western blots images are shown. a - difference vs NC ($p < 0.05$); b - difference vs D1KD ($p < 0.05$); c - difference vs D4KD ($p < 0.05$); * $p < 0.05$ significantly different from visADMSCs. Designation of the groups: NC – negative control containing non-targeting siRNA fragment; D1KD – knockdown of TBC1D1; D4KD – knockdown of TBC1D4; D1/D4KD – double knockdown of TBC1D1 and TBC1D4.

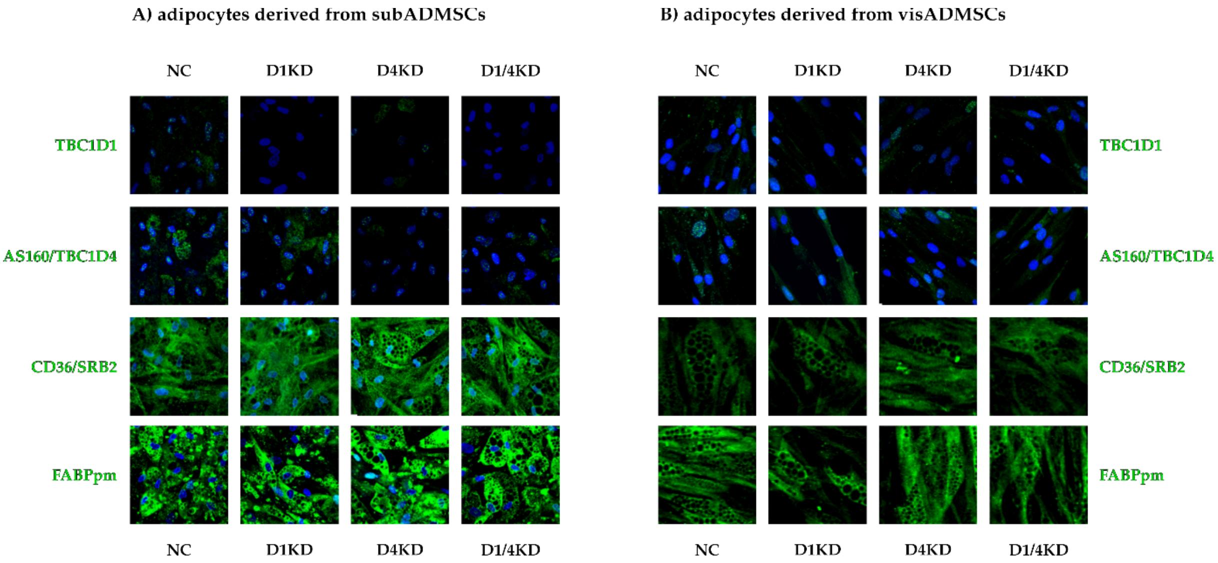

Figure S5. Intracellular localization of RabGTPase-activating proteins (TBC1D1 and TBC1D4) and FA transporters (CD36/SR-B2, FABPpm) in the adipocytes differentiated from (**A**) sub- and (**B**) visADMSCs was analyzed by the means of confocal microscopy.

Tables

	Lean subjects (n = 4)
Gender	Female (n = 4)
Age [years]	59.25 ± 5.737
Waist [cm]	75.25 ± 4.646
WHR	0.78 ± 0.023
CRP [mg/l]	5.55 ± 0.412
Glucose [mg/dl]	72 ± 3.559
Insulin [μIU/ml]	7.65 ± 0.714
HOMA-IR	1.36 ± 0.133
ALT [IU/l]	24.75 ± 4.992
AST [IU/l]	22.25 ± 4.5
Chol [mg/dl]	172.75 ± 7.848
LDL [mg/dl]	115.5 ± 0.577
TG [mg/dl]	127 ± 6.782
HDL [mg/dl]	65.5 ± 1.291
WBC [10^3/μl]	7.73 ± 1.307
RBC [10^6/μl]	4.9 ± 0.245
HGB [g/dl]	13.95 ± 0.311
PLT [10^3/μl]	292.75 ± 7.932
fibrynogen [mg/dl]	373.5 ± 13.964
INR	0.87 ± 0.039
BP systolic	125 ± 10
BP diastolic	81.25 ± 6.292

Table S1. The anthropometric and clinical characteristics of donors.

ALT – alanine transaminase; AST – asparatate transaminase; CRP – C-reactive protein; HDL – high density lipoprotein; HGB – hemoglobin; HOMA-IR – homeostatic model assessment of insulin resistance; INR – international normalized ratio; LDL – low-density lipoprotein; PLT – platelet count; RBC – red blood cell count; TAG – triacylgycerol; WBC – white blood cell count; WHR – waist-hip ratio. Basic characteristic of patients (tissue donors). Data given as mean ± SD.
